# CRISPR/Cas13a Powered Portable Electrochemiluminescence Chip for Ultrasensitive and Specific MiRNA Detection

**DOI:** 10.1002/advs.201903661

**Published:** 2020-05-27

**Authors:** Ting Zhou, Ru Huang, Mengqi Huang, Jinjin Shen, Yuanyue Shan, Da Xing

**Affiliations:** ^1^ MOE Key Laboratory of Laser Life Science & Institute of Laser Life Science College of Biophotonics South China Normal University Guangzhou 510631 China

**Keywords:** CRISPR/Cas13a, light‐switch [Ru(phen)_2_dppz]^2+^, miRNA detection, pBPE‐ECL, *trans*‐cleavage activity

## Abstract

MicroRNAs (miRNAs) have been widely investigated as potential biomarkers for early clinical diagnosis of cancer. Developing an miRNA detection platform with high specificity, sensitivity, and exploitability is always necessary. Electrochemiluminescence (ECL) is an electrogenerated chemiluminescence technology that greatly decreases background noise and improves detection sensitivity. The development of a paper‐based ECL biosensor further makes ECL suitable for point‐of‐care detection. Recently, clustered regularly interspaced short palindromic repeats (CRISPR)/Cas13a as high‐fidelity, efficient, and programmable CRISPR RNA (crRNA) guided RNase has brought a next‐generation biosensing technology. However, existing CRISPR/Cas13a based detection often faces a trade‐off between sensitivity and specificity. In this research, a CRISPR/Cas13a powered portable ECL chip (PECL‐CRISPR) is constructed. Wherein target miRNA activates Cas13a to cleave a well‐designed preprimer, and triggers the subsequent exponential amplification and ECL detection. Under optimized conditions, a limit‐of‐detection of 1 × 10^−15^ m for miR‐17 is achieved. Through rationally designing the crRNA, the platform can provide single nucleotide resolution to dramatically distinguish miRNA target from its highly homologous family members. Moreover, the introduction of “light‐switch” molecule [Ru(phen)_2_dppz]^2+^ allows the platform to avoid tedious electrode modification and washing processes, thereby simplifying the experimental procedure and lower testing cost. Analysis results of miRNA from tumor cells also demonstrate the PECL‐CRISPR platform holds a promising potential for molecular diagnosis.

## Graphical abstract

A clustered regularly interspaced short palindromic repeats (CRISPR)/Cas13a powered portable pBPE‐ECL chip (PECL‐CRISPR) is developed, which combines the high‐fidelity and *trans*‐cleavage of Cas13a, high efficiency of exponential amplification, high sensitivity and low background noise of electrochemiluminescence technology, and the simplicity of “light‐switch” [Ru(phen)_2_dppz]^2+^, thereby enabling ultrasensitive, highly‐specific, convenient, and low‐cost detection of miRNA.

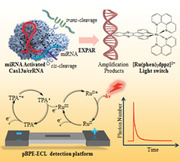

## 1 Introduction

Mature microRNAs (miRNAs) are small noncoding RNAs (usually 18–23 nucleotides (nts)) that regulate protein‐coding gene expression by repressing translation or cleaving RNA transcripts.^[^ ^1^ ^]^ The aberrant expression of miRNA has been confirmed involve in many kinds of tumors. Thus, miRNA‐expression profiles can be served as biomarkers in cancer diagnosis, prognosis, and response to treatment.^[^ ^2^ ^]^ Although numerous researchers have devoted to develop accurate miRNA detection methods, it is still challenging due to the unique characteristics of miRNA, such as small size, low cellular abundance, and high homology.^[^ ^3^ ^]^ Therefore, developing ultrasensitive and specific approach for miRNA detection is always necessary.

CRISPR (Clustered regularly interspaced short palindromic repeats)‐Cas (CRISPR‐associated) systems are originally derived from prokaryotic adaptive immune system against invading nucleic acid components.^[^ ^4^ ^]^ Recent years, CRISPR‐Cas system as an efficient gene editing tool attracted a large number of interests of biological researchers.^[^ ^5^ ^]^ Generally, CRISPR/Cas system can divide into two main classes, class I and II, according to the system comprise a single or multiple effector.^[^ ^6^ ^]^ Among them, class II (e.g., Cas9, Cas12, and Cas13) possesses more widely application due to its simple components (a single effector protein and a programmable guide RNA).^[^ ^7^ ^]^ Especially, the newly founded *trans* cleavage activity of Cas12a, Cas13a, and Cas14a made CRISPR/Cas system an ideal candidate of next‐generation diagnostic biosensing platforms.^[^ ^8^ ^]^ **Table** **1** displays existing CRISPR/Cas based nucleic acid detection methods, and compares their features and performances. Among them, CRISPR/Cas13a that belonging to Class II type VI‐A CRISPR/Cas system is the only RNA targeting CRISPR effector, and possesses transcendent signal amplification ability.^[^ ^9^ ^]^ In Brief, Cas13a and its corresponding CRISPR RNA (crRNA) can specifically recognize and cleavage target RNA based on the complementarity between crRNA and target,^[^ ^10^ ^]^ followed by activate itself *trans* cleavage activity to collaterally cleave the fluorophore‐quencher labeled RNA probes with a turnover efficiency of ≈4854,^[^ ^11^ ^]^ thereby enables signal amplified fluorescence detection. Through this method, a limit‐of‐detection of 1 × 10^−12^ m for viral RNA can be achieved.^[^ ^10^, ^11^ ^]^ To further improve the sensitivity, some amplification strategies, such as recombinase polymerase amplification (RPA), have been introduced into CRISPR/Cas system.^[^ ^5^, ^12^ ^]^ However, Cas protein in these methods always used as an efficient nuclease to recognize the presupposed amplification products, thus its high‐fidelity and programmable RNA recognition ability were unfortunately ignored. Therefore, create a new detection strategy that combined the high specificity and signal amplification ability of CRISPR/Cas system with great efficiency of exponential nucleic acid amplification should be valuable.

**Table 1:** Comparison of existing CRISPR/Cas based nucleic acid detection methods

System name	Effector^a)^	Detecting technique	Amplification	LOD	Detection ranges	SBD	Target
One‐step detection^[^ ^11^ ^]^	LbuCas13a	Fluorescence	—	450 × 10^−15^ m	100 × 10^−15^ m–10 × 10^−15^ m	Yes	miRNA
RACE^[^ ^8e^ ^]^	spyCas9	Fluorescence	RCA	90 × 10^−15^ m	1 × 10^−12^ m–10 × 10^−9^ m	NA	miRNA
SHERLOCK^[^ ^5a^ ^]^	LwaCas13a	Fluorescence	RPA	2 × 10^−3^ × 10^−15^ m	0.01 × 10^−18^ m–10 × 10^−9^ m	Yes	DNA/RNA
CASLFA^[^ ^12d^ ^]^	Cas9	Colorimetry	PCR/RPA	150 copies	200–2 × 10^8^ copies	Yes	DNA
NE‐CRISPR^[^ ^12e^ ^]^	AsCas12a/LbuCas13a	Colorimetry	RPA/PCR	200 copies 500 × 10^−15^ m	200–2 × 10^7^ copies 500 × 10^−15^ m–10 × 10^−9^ m	Yes	DNA miRNA
E‐CRISPR^[^ ^7e^ ^]^	AsCas12a	Electrochemical	—	50 × 10^3^ × 10^−15^ m	10 × 10^−15^ m–100 × 10^−9^ m	Yes	DNA
EM‐CRISPR^[^ ^8d^ ^]^	LwaCas13a	Electrochemical Microfluidic	—	10 × 10^3^ × 10^−15^ m	10 × 10^−15^ m–1 × 10^−9^ m	Yes	miRNA
PECL‐CRISPR	LbuCas13a	ECL	EXPAR	1 × 10^−15^ m	1 × 10^−15^ m–100 × 10^−15^ m	Yes	miRNA

a) LbuCas13a: *Leptotrichia buccalis* Cas13a; LwaCas13a: *Leptotrichia wadei* Cas13a; AsCas12a: *Acidaminococcus sp* Cas12a; SpyCas9: *Streptococcus pyogenes* Cas9; LOD: limit‐of‐detection; SBD: single base discrimination; NA: not available; RPA: recombinase polymerase amplification; RCA: rolling circle amplification.

Electrochemiluminescence (ECL) is a electrochemistry triggered chemiluminescence techniques, which combines the advantages of chemiluminescence and electrochemistry, such as low background noise and wide dynamic range.^[^ ^13^ ^]^ Bipolar electrode (BPE) is an electrical conductor material in electrolyte solution without direct electrical connection to external direct‐current (DC) power.^[^ ^13^, ^14^ ^]^ Such simplicity enables BPE easy to integrate with portable device. Accordingly, paper‐based BPE (pBPE) ECL biosensor has attracted considerable attention for biosensing characteristic gene of pathogens,^[^ ^15^ ^]^ protein,^[^ ^16^ ^]^ and metal ions^[^ ^17^ ^]^ due to the high sensitivity, low‐cost, and portability.^[^ ^18^ ^]^

In this article, we first introduce CRISPR/Cas13a into pBPE‐ECL biosensing platform (PECL‐CRISPR) for ultrasensitive and specific miRNA detection. Briefly, Cas13a was utilized to directly recognize miRNA target and collaterally cleave a well‐designed pretrigger (PT) into mature‐trigger (MT), which can be as a primer to mediate the subsequent exponential amplification. Different from previous Cas13a based RNA detection methods, our strategy takes full advantages of the specific RNA recognition and signal amplification abilities of Cas13a. Both the *trans* cleavage activity of Cas13a and isothermal exponential amplification (EXPAR) can contribute the detection sensitivity.^[^ ^19^ ^]^ Meanwhile, single base resolution can be achieved by flexibly designing the guide sequence of crRNA. Cost‐ and labor‐effective pBPE platform was selected to perform ECL detection. Furthermore, the introduction of light‐switch [Ru(phen)_2_dppz]^2+^ makes the detection platform avoiding tedious electrode modification and washing processes, thereby simplify experimental procedure, lower testing cost, as well as improve the reproducibility. In addition, the application ability of PECL‐CRISPR for the detection of miRNA in raw cell lysates was also evaluated.

## 2 Results

### 2.1 Principle of the PECL‐CRISPR miRNA Profiling Platform

**Scheme 1:**
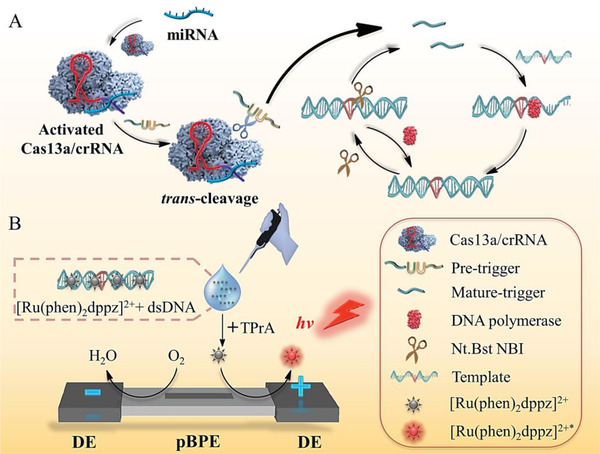
A,B) Principle scheme of CRISPR/Cas13a powered portable ECL (PECL‐CRISPR) chip for ultrasensitive and specific miRNA detection.

**Scheme** **1** shows an overall illustration of PECL‐CRISPR miRNA detection platform and how it works. miR‐17 that involves many diseases, including breast cancer, pulmonary hypertension, and Mantle cell lymphoma was selected as a model target for proof‐of‐concept.^[^ ^20^ ^]^ The Cas13a used in this research is from *Leptotrichia buccalis* (LbuCas13a). The crRNA consisting of a 30 nt repeat region for interaction with Cas13a and a 20 nt programmable guide region (termed spacer) for target RNA recognition. Considering the *trans* cleavage activity of LbuCas13a is more prefer to cleave Uracil flanked ribose‐phosphodiester bond,^[^ ^12b^ ^]^ we designed a PT containing two Uracil ribonucleotides (rU), 5′‐end of which possesses 16 complementary bases with the amplification template and five random bases in the other end. The amplification template is designed with a central nicking endonuclease (NEase) acting site and two separated repeat sequences, both of which are complementary to the 5′ end of the PT. In addition, both the PT and the amplification template was added a C3 spacer at the 3′‐end to avoid target‐independent polymerase amplification.^[^ ^21^ ^]^

First, assembled Cas13a/crRNA system specifically recognize and cleave target miRNA, thereby trigger its own *trans* cleavage activity by moving the two conserved higher eukaryotes and prokaryotes nucleotide binding (HEPN) domains of Cas13a to form a new RNase active domain. Next, Cas13a *trans* cleave the PT into two fragments, in which 5′ fragment, named MT can hybridize with the amplification template and initiate polymerization reaction in the presence of T4 PNK and Vent DNA polymerase. The newly formed dsDNA is recognized and nicked by the NEase. Through the nicking and strand extension/displacement cycle, large amounts of dsDNA products can be generated. Relatively, the Cas13a *trans*‐cleavage activity is silenced in the absence of the target, and the noncleaved PT cannot be extended by DNA polymerase and trigger downstream EXPAR.

Then, the “light switch” [Ru(phen)_2_dppz]^2+^ was added into the Cas13a mediated EXPAR (CAS‐EXPAR) system. Dissociative [Ru(phen)_2_dppz]^2+^ cannot produce luminescence signal due to the protonation of N atoms in aqueous solution. After interacting with the dsDNA products of EXPAR, the luminescence signal can be greatly increased due to the planar phenazine ligand of [Ru(phen)_2_dppz]^2+^ can interact with the base pairs in the major groove of dsDNA, and render the N atoms of phenazine in a protected state, thus improve the population of luminescent state. Subsequently, the [Ru(phen)_2_dppz]^2+^ added amplification system and excess TPrA were introduced into the pBPE‐ECL platform. The ECL signal can be collected through photomultiplier tube (PMT) when DC power supplies the driving voltage. The reaction mechanism between [Ru(phen)_2_dppz]^2+^‐DNA complex and coreactant TPrA is as follows^[^ ^15b^ ^]^

(1) $$\begin{array}{c} {\left[\mathrm{Ru}{\left(\mathrm{phen}\right)}_{2}\mathrm{dppz}\right]}^{2+}-\mathrm{DNA}-e^{-}\to {\left[\mathrm{Ru}{\left(\mathrm{phen}\right)}_{2}\mathrm{dppz}\right]}^{3+}-\mathrm{DNA} \end{array}$$

(2) $$\begin{array}{c} \mathrm{TPrA}-e^{-}\to \mathrm{TPr}A^{•+}\to \mathrm{TPr}A^{•}+H^{+} \end{array}$$

(3) $$\begin{array}{ccc} & & {\left[\mathrm{Ru}{\left(\mathrm{phen}\right)}_{2}\mathrm{dppz}\right]}^{3+}-\mathrm{DNA}+\mathrm{TPr}A^{•}\to {{\left[\mathrm{Ru}{\left(\mathrm{phen}\right)}_{2}\mathrm{dppz}\right]}^{2+}}^{*}\\ & & \;-\mathrm{DNA}+\mathrm{products} \end{array}$$

(4) $$\begin{array}{c} {{\left[\mathrm{Ru}{\left(\mathrm{phen}\right)}_{2}\mathrm{dppz}\right]}^{2+}}^{*}-\mathrm{DNA}\to {\left[\mathrm{Ru}{\left(\mathrm{phen}\right)}_{2}\mathrm{dppz}\right]}^{2+}-\mathrm{DNA}+hv \end{array}$$

In this research, the ECL signals on BPE directly indicate the oxidation reaction of [Ru(phen)_2_dppz]^2+^‐DNA complex at the anode of BPE and is proportional to target miRNA concentration, thereby enables quantitative miRNA detection.

### 2.2 Characterization of the pBPE‐ECL Chip

The size of each part of the well‐designed pBPE was shown in Figure S1A (Supporting Information). The pBPE chip and the assembled pBPE chip were shown in Figure S1B,C (Supporting Information), respectively. The boundary of wax pattern, the front face of wax‐penetrated paper, as well as the carbon ink‐screen‐printed paper were characterized by SEM (Figure S1D–F, Supporting Information, respectively), which can confirm the successful preparation of the pBPE chip.

### 2.3 Evaluation of *trans*‐Cleavage Activity of CRISPR/Cas13a

**Figure 1:**
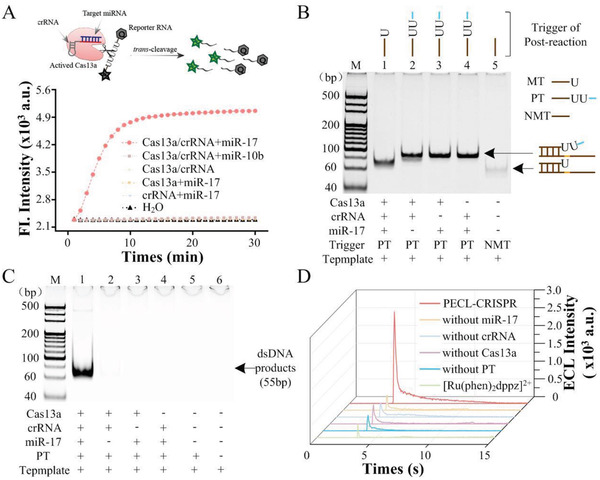
Feasibility analysis of PECL‐CRISPR system for miRNA detection. A) Real‐time fluorescence assay of miR‐17 activated Cas13a/crRNA *trans* cleavage. FQ5U was served as the reporter. B) Nondenaturing PAGE analysis of the cleavage ability of Cas13a/crRNA for rU‐bearing ssDNA. NMT is a ssDNA without rU. C) PAGE analysis of the CAS‐EXPAR products by SYBR Green I staining and nondenaturing PAGE. M is marker. The concentration of the PT and the template were 5 × 10^−9^ m and 100 × 10^−9^ m, respectively. D) ECL analysis of PECL‐CRISPR system. 1 × 10^−9^ m miR‐17 was used in these experiments. All the ECL results from the pBPE collected by a PMT with 0.5 × 10^−3^ m [Ru(phen)_2_dppz]^2+^, 70 × 10^−9^ m TPrA, pH 7.4, and the driving voltages was 14 V.

The purity of the LbuCas13a protein analyzed by sodium dodecyl sulfate‐polyacrylamide gel electrophoresis (PAGE) and Coomassie blue staining was estimated to be 95% (Figure S2A, Supporting Information). Owing to LbuCas13a prefer to cleave Uracil ribonucleotide (rU) flanked phosphodiester bond, a five‐rU consisted and FAM/BHQ1 double‐labeled ssRNA reporter (FQ5U) was used to real‐time fluorescence monitoring of the *trans* cleavage activity of Cas13a/crRNA. As shown in **Figure** **1**A, the fluorescence intensity of sample with Cas13a/crRNA complex and miR‐17 increased rapidly and reached saturation within 10 min. Relatively, the fluorescent signal barely changed without Cas13a, crRNA, or target miRNA. Also nontarget miRNAs (such as miR‐10b) cannot cause fluorescence enhancement, which confirmed the Cas13a/crRNA system only can be activated by miR‐17. All of the above results indicated that the LbuCas13a/crRNA complex can accurately recognize target miRNA and initiated its *trans* cleavage activity to hydrolysis the nontarget RNA reporter.

Then, whether Cas13a/crRNA could effectively cleave rU‐bearing DNA was examined by 10% PAGE. As shown in Figure 1B, the hybrids of the template and the Cas13a/crRNA *trans*‐cleavage produced MT (Lane 1) showed significantly lower band than that of PT‐template hybrids (Lane 2–4), thus indicated that the activated Cas13a/crRNA can effectively cleave the PT into MT, which can further hybridize to the template for the following amplification circuit. Moreover, to test whether the trans‐cleavage activity of Cas13a would be affected by the number of rU in the substrate, a reporter probe (length of 5 nt) named FQ2U was designed to have two rU in the middle, as well as a FAM dye and a BHQ1 quencher at the two ends, respectively. Different concentrations of FQ2U (0.001, 0.01, 0.1, 0.2, 0.5, 1, 2, 5, and 10 × 10^−6^ m) were incubated with 10 × 10^−9^ m LbuCas13a/crRNA complex and 1 × 10^−9^ m miR‐10b, and tested by real‐time fluorescence monitoring. The results were shown in Figure S3A (Supporting Information). The Michaelis constant of LbuCas13a (*K*_m_) was determined by plotting initial velocity (*V*_0_) as a function of substrate concentration ([S]) (Figure S3B). The Michaelis–Menten equation can be obtained according to the equation of *V*_0_ = (*V*_max_[S])/(*K*_m_+[S]), wherein *V*_max_ is maximum reaction rate. The turnover number of LbuCas13a (*k*_cat_) was calculated by the equation of *k*_cat_ = *V*_max_/*E*_t_ (*E*_t_ indicating the concentration of LbuCas13a is 10 × 10^−9^ m) as ≈204. The catalytic efficiency *k*_cat_/*K*_m_ was 0.77854 × 10^8^ s^−1^ M^−1^, which is > 1400‐fold lower than using five consecutive rU as the *trans*‐cleavage substrate of LbuCas13a (*k*_cat_/*K*_m_ of 1.09 × 10^9^ s^−1^ M^−1^, *E*_t_ was 0.01 × 10^−9^ m) that we reported previously.^[^ ^1^ ^]^ Overall, decreasing the rU number of the DNA oligo can significantly lower the *trans*‐cleavage efficiency of LbuCas13a/crRNA. Also, we attempted to use LbuCas13a cleaved preprimer to hybridize with cyclized padlock probe and execute rolling circle amplification (RCA). The PAGE result (Figure S4, Supporting Information) showed that the cleaved preprimer cannot trigger RCA reaction directly. According to previous report, Cas13 *trans*‐cleaved 5′‐fragment possesses a 2′,3′‐cyclic phosphate at its 3′‐end,^[^ ^12b^ ^]^ which may hinder the polymerization reaction, thus T4 polynucleotide kinase (T4 PNK) was used to transform the 2′, 3′‐cyclic phosphate group into 3′‐phosphate group. The PAGE image of Figure S4 (Supporting Information) showed that T4 PNK‐treated Cas13a‐cleaved preprimer can induce obvious RCA products, which indicated that T4 PNK mediated phosphorylation is the key to initiate DNA polymerization reaction.

### 2.4 Feasibility of PECL‐CRISPR Platform for miRNA Detection

Next, the EXPAR system was analyzed by PAGE. As shown in Figure 1C, only the sample with 1 × 10^−9^ m miR‐17 showed obvious dsDNA products band (55 bp, Lane 1). Relatively, no band was observed in the samples without miRNA, crRNA, Cas13a, or PT. These results demonstrated that the EXPAR can only be initiated by target miRNA. Furthermore, the Cas13a mediated EXPAR (CAS‐EXPAR) was measured with pBPE‐ECL platform. The results of ECL measurements (Figure 1D) are consistent with those of PAGE. Moreover, the ECL signal reached the maximum at 10 s and gradually decays over time. Above all, the PECL‐CRISPR platform can be applied for miRNA detection.

### 2.5 Optimization of the Reaction Conditions

To obtain better performance, a series of experimental conditions of PECL‐CRISPR were optimized. First, the concentration of Cas13a/crRNA would directly influence the *trans*‐cleavage efficiency for PT, thereby affects the amplification efficiency of subsequent EXPAR. Different concentrations (2.5 × 10^−9^ m, 5 × 10^−9^ m, 10 × 10^−9^ m, and 20 × 10^−9^ m, respectively) of Cas13a/crRNA with a same ratio of 1:1 were introduced into CAS‐EXPAR system and analyzed by PAGE. As can be seen from Figure S5A (Supporting Information), 10 × 10^−9^ m Cas13a/crRNA exhibited the greatest ratio of signal to background (S/B) of 13.306. However, when the concentration of Cas13a/crRNA increased to 20 × 10^−9^ m, a target‐independent band appeared, which reflected excess Cas13a/crRNA may cause nonspecific cleavage of PT. Hence, 10 × 10^−9^ m Cas13a/crRNA was applied for the following PECL‐CRISPR profiling. Another key factor to the PECL‐EXPAR performance is the concentration of PT. Different concentrations of PT in response to a same target concentration of 1 × 10^−9^ m were also tested by PAGE. The results (Figure S5B, Supporting Information) showed that 50 × 10^−9^ m PT can achieve the highest S/B of 15.323. Relatively, insufficient PT (≤ 25 × 10^−9^ m) could not support a complete reaction, while excess PT (100 × 10^−9^ m) would cause the increase of background signal. Hence, the concentration of PT was identified to be 50 × 10^−9^ m for PECL‐CRISPR profiling.

Futhermore, to test the effect of the concentration of [Ru(phen)_2_dppz]^2+^ to the performance of PECL‐CRISPR for miRNA detection, different concentration of [Ru(phen)_2_dppz]^2+^ were added into the PECL‐CRISPR system, respectively. The result was shown in Figure S6 (Supporting Information). It can be seen that the signal‐to‐background ratio (*E*_T_/*E*_C_) reached maximum (≈5.3) when [Ru(phen)_2_dppz]^2+^ was 0.3 × 10^−3^ m. With the further increase of [Ru(phen)_2_dppz]^2+^ concentration, the *E*_T_/*E*_C_ gradually decreased due to the growth of the background signal. Therefore, 0.3 × 10^−3^ m [Ru(phen)_2_dppz]^2+^ was selected as the optimal concentration.

### 2.6 Sensitivity Analysis of PECL‐CRISPR

**Figure 2:**
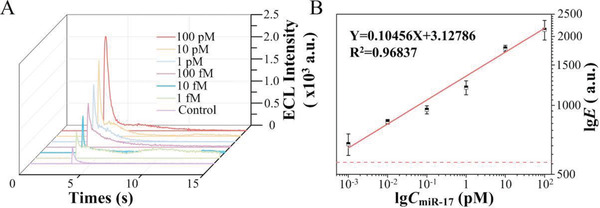
Sensitivity analysis of PECL‐CRISPR system. A) The analysis results of PECL‐CRSIPR for various concentrations of target miR‐17 (100 × 10^−12^ m, 10 × 10^−12^ m, 1 × 10^−12^ m, 100 × 10^−15^ m, 10 × 10^−15^ m, and 1 × 10^−15^ m, respectively). Control sample is the negative sample without miR‐17. B) Linear analysis of the miR‐17 detection results by the PECL‐CRISPR system. The red dashed line represents the detection limit. Error bars are the standard deviation of triplicate measurements.

For the PECL‐CRISPR platform, both the *trans*‐cleavage activity of Cas13a and EXPAR can contribute the signal amplification. According to previous reports, LbuCas13a *trans* cleavage activity and EXPAR can provide about 10^3^‐fold and 10^6^–10^9^‐fold efficiency of signal amplification, respectively.^[^ ^11^, ^19^ ^]^ To evaluate the sensitivity of PECL‐CRISPR platform for miR‐17 quantitation, a series of concentrations of miR‐17 (ranging from 1 × 10^−15^ m to 100 × 10^−12^ m) were measured under the optimal conditions. As shown in **Figure** **2**A, a gradual increase of ECL intensity was observed as the miR‐17 concentration increased. The logarithms of ECL intensity was linearly related to the logarithms of miR‐17 concentration with a correlation equation of lg*E* = 3.12 786 + 0.10 456 lg*C*_miR‐17_ (× 10^−12^ m), and the R^2^ can be calculated to 0.96 837, and the relative standard deviation (RSD%) of measurement for three batches of these samples were in the range of 2.5–12.5%, which reflected the PECL‐CRISPR chip holds a good stability (Figure 2B). The limit of detection (defined as the concentration of target that yields a net signal equivalent to three times the standard deviation of three replicates of the control sample without target) can reach 1 × 10^−15^ m. Compared to the previous studies about LbuCas13a mediated direct detection of miR‐17 (limit‐of‐detection (LOD) is 1 × 10^−12^ m),^[^ ^11^ ^]^ the detection limit of PECL‐CRISPR system was ≈3 orders of magnitude lower.

Also, the LOD of Cas13a/crRNA and CAS‐EXPAR were compared by real‐time fluorescence monitoring. First, a 5‐nt FAM and BHQ1 labeled probe bearing two rU (FQ2U) was used as the reporter of Cas13a/crRNA mediated miRNA detection. The experimental results are shown in Figure S8A (Supporting Information). Relatively, the CAS‐EXPAR was evaluated by real‐time fluorescence monitoring with SYBR Green I as the reporter, and the results are shown in Figure S8B (Supporting Information). From the results, the LOD of Cas13a/crRNA for miR‐17 detection was calculated to 100 × 10^−12^ m, and the LOD of CAS‐EXPAR was calculated to 27 × 10^−15^ m. Thus, EXPAR can improve the LOD by up to 3700‐fold.

### 2.7 Specificity Analysis of PECL‐CRISPR

Except flexible programmability of crRNA and efficient signal amplification efficiency of Cas13a, high selectivity is another great advantage of Cas13a/crRNA system. Direct Cas13a/crRNA based nucleic acid detection systems have been confirmed possess single base resolution, while often limit by the sensitivity of  × 10^−15^ m level.^[^ ^8^, ^10^, ^11^ ^]^ Although many amplification methods, such as RPA, were introduced into Cas13a/crRNA system for improving detection sensitivity,^[^ ^5^, ^12^ ^]^ they only used Cas13a as an efficient RNase to recognize presupposed amplification products, while the specific recognition of target is depended on other enzyme (recombinase in this case). In this research, Cas13a/crRNA first recognized target RNA and mediated efficient cleavage of PT, which could then trigger the following amplification reaction. Therefore, the CAS‐EXPAR system not only contributes to the sensitivity, but also plays a key role on the specific recognition of target.

**Figure 3:**
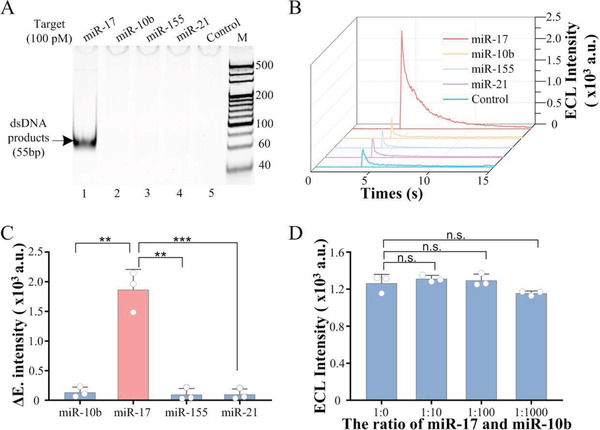
Specificity analysis of PECL‐CRISPR system. A) Nondenaturing PAGE analysis results of CAS‐EXPAR with 100 × 10^−12^ m different miRNAs (miR‐17, miR‐10b, miR‐155, and miR‐21, respectively). B) ECL detection results of PECL‐CRISPR system for 100 × 10^−12^ m different miRNAs. C) T test of the maximum of the ECL signal intensity for each miRNA. (two‐tailed Student *t*‐test; **P < 0.01; ***P < 0.001; All plots show mean ± SD for *n* = 3 replicates). D) ECL detection results for the samples mixing miR‐17 and miR‐10b with different ratios (1:0, 1:10, 1:100, and 1:1000) (two‐tailed Student *t*‐test; n.s., not significant; **P < 0.01; ***P < 0.001; All plots show mean ± SD for *n* = 3 replicates).

The specificity of PECL‐CRISPR system was first investigated by, respectively, adding four different miRNAs (miR‐17, miR‐10b, miR‐155, and miR‐21) into the CAS‐EXPAR system and analyzed by 10% PAGE. The results (**Figure** **3**A) showed the dsDNA products can only be observed in the sample with miR‐17. Also, the results were verified using PECL‐CRISPR platform (Figure 3B). *t* test was performed on the Δ*E* values of miR‐17 and other three miRNAs with a same concentration of 100 × 10^−12^ m (*P* < 0.01) (Figure 3C), wherein the value of Δ*E* is the net signal that calculated by subtracting the signal of negative control from the total signal. It is indicated that PECL‐CRISPR system can dramatically distinguish miR‐17 from other three miRNAs. Furthermore, a series of samples that mixed miR‐17 and miR‐10b with different molar ratio (1:0, 1:10, 1:100, and 1:1000) were also tested. The results (Figure 3D) showed there was no significant difference in ECL intensity (*P* > 0.05), which demonstrated that the system hold a potential for analyzing clinically complex sample.

**Figure 4:**
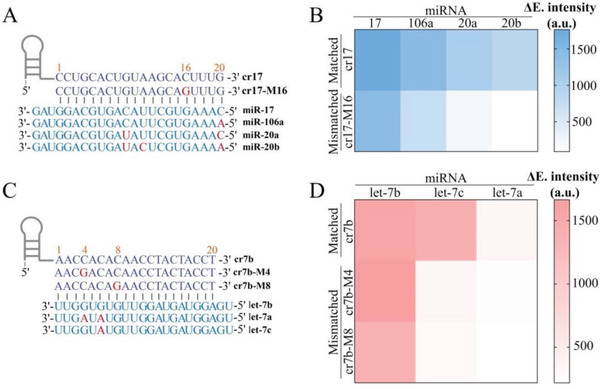
Specificity analysis of PECL‐CRISPR for miR‐17 and let‐7 family members. A) Sequences of the crRNA with or without mismatch, as well as the miR‐17 family members. Mismatched/different nucleotides in crRNA and target miRNA are marked in red. B) Heat map analysis of the PECL‐CRISPR detection results for four homologous miR‐17 family members. C) Sequences of crRNA with or without mismatch, as well as the let‐7 family members. Mismatched/different nucleotides in crRNA and target miRNA are marked in red. D) Heat map analysis of the PECL‐CRISPR detection results for three homologous let‐7 family members. The intensity value is the average of the results from three different batches of PECL‐CRISPR experiments.

To further explore the specificity of PECL‐CRISPR system, four similar miR‐17 family members, miR‐17, miR‐106a, miR‐20a, and miR‐20b, were detected using PECL‐CRISPR platform. According to the previous reports, a “bubble” or distortion on crRNA/target RNA hybrid can impact the conformation of LbuCas13a/crRNA/target complex and further impede the *trans*‐cleavage activity of Cas13a in a number and position‐dependent manner.^[^ ^5^, ^10^ ^]^ In this experiment, a mismatch was intentionally introduced into the 16 position of the crRNA spacer to reduce the mismatch tolerance of Cas13a/crRNA (**Figure** **4**A). The fluorescence analysis results were shown in Figure 4B. Compared with perfectly matched crRNA (cr17), the crRNA with a mismatch (cr17‐M16) can provide higher selectivity. Especially, miR‐106a also can be dramatically differentiated from miR‐17 by Cas13a/cr17‐M16, even there is only a single base variation at the 5′ end of the miRNAs. Relatively, the crRNA perfectly matched with miR‐17 contributed similar signal intensity for miR‐106a. Therefore, the introduction of mismatch at the 16 position of the crRNA spacer can greatly improve the specificity.

To further verify the specificity and universality of PECL‐EXPAR, the highly conserved let‐7 family miRNAs, let‐7a, let‐7b, and let‐7c were also, respectively, introduced into PECL‐CRISPR platform. Both of let‐7a and let‐7c possess two or one base variation at the 3′‐end compared to let‐7b (Figure 4C). According to the published conclusions, the targets with mismatched base(s) that corresponding to the 5–8 site of crRNA spacer could hinder the HEPN‐nuclease activity of LbuCas13a, although the crRNA still had a high affinity with target RNA. Moreover, continuous base mismatches would form a larger “bubble” and further reduce the mismatch tolerance of Cas13a/crRNA. Thus, two crRNAs were, respectively, designed with a mismatch at the 4 or 8 site of the spacer (cr7b‐M4 and cr7b‐M8), which are adjacent to the different base(s) among the let‐7 family members. The results showed both of the crRNAs exhibited greater selectivity for single‐base distinction over perfectly matched crRNA (cr7b) (Figure 4D), and the discrimination ability of cr7b‐M8 for let‐7 family members was higher than cr7b‐M4. More importantly, the above results confirmed that a single base resolution can be achieved by flexibly introducing a mismatch at the specific site of crRNA, no matter the single different base locates at the 5′ or 3′ end of the target.

### 2.8 Analyzing the Performance of PECL‐CRISPR for the Detection of MiR‐17 in Cell Extracts and Raw Cell Lysates

**Figure 5:**
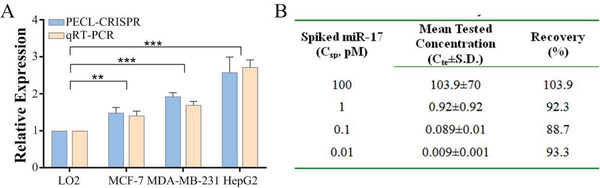
Analyzing the performance of PECL‐CRISPR for the detection of miR‐17 in cell extracts and raw cell lysates, respectively. A) Comparison of the detection results of PECL‐CRISPR and qRT‐PCR for the relative expression of miR‐17 in different cell lines (two‐tailed Student *t*‐test; n.s., not significant; **P < 0.01; ***P < 0.001; All plots show mean ± SD for *n* = 3 replicates). B) Analysis of the test results of PECL‐CRISPR for different concentration of miR‐17 in raw LO2 cell lysates. Mean represents the average of the net ECL signal (the total ECL signal minus the background signal) from three batches of experiment.

The application ability of PECL‐CRISPR was investigated by detecting miR‐17 from different human tumor cells, including human breast adenocarcinoma cells (MDA‐MB‐231 and MCF‐7) and human hepatocellular liver carcinoma cell lines (HepG2). Human normal liver cell (LO2) was selected as the control sample. Quantitative real‐time polymerase chain reaction (qRT‐PCR) was used as the standard method to validate the accuracy of PECL‐CRISPR. 300 ng of total small RNA was extracted from the cells and added to the reaction system. As shown in **Figure** **5**A, the relative expression of miR‐17 in all of the three tumor cells displayed varying degrees of increase compared to the LO2 (two‐tailed Student *t*‐test; MCF7: *P* < 0.01; MDA‐MB‐231 and HepG2: *P* < 0.001), which are consistent with qRT‐PCR analysis results (Figure S9, Supporting Information). For the qRT‐PCR, small nuclear RNA (snRNA) U6 was used as the generic endogenous contrast, and the computation formula for relative expression is as follows: Fold change = 2^−ΔΔ^ *^C^* ^t^. The probe sequence and the *C*_t_ value of qRT‐PCR are shown in Tables S2 and S3 (Supporting Information).

Moreover, the application ability of PECL‐CRISPR for the detection of miR‐17 in cell lysates was also evaluated. Briefly, different concentration of miR‐17 were spiked into raw cell lysate, which was obtained by thermal lysing LO2 cells and analyzed by PECL‐CRISPR. The detection results were shown in Figure 5B. The recovery (%) was defined as the ratio of the mean tested concentration (*C*_te_) by PECL‐CRISPR and the concentration of the spiked miR‐17. Wherein, *C*_te_ was obtained by substituting the net ECL signal value (the total ECL signals subtract the signal of the control sample without added miR‐17) into the standard curves. From the results, it can be seen that the PECL‐CRISPR detection platform possesses certain reproducibility. The standardization and automation of the preparation process of pBPE‐ECL platform will help to further improve the stability of the detection.

## 3 Conclusion

In this study, we developed a CRISPR/Cas13a powered portable ECL chip (named PECL‐CRISPR) for ultrasensitive and high‐specific analysis of miRNA. Compared to previous Cas13a based signal amplification strategies, the PECL‐CRISPR platform not only utilizes the *trans* cleavage activity of CRISPR/Cas13a to mediate subsequent exponential amplification for dramatically improving the detection sensitivity, but takes full advantage of the specific RNA recognition ability of Cas13a to achieve single base resolution. Under optimized conditions, the platform can provide a limit‐of‐detection of 1 × 10^−15^ m for miR‐17. More importantly, the platform can distinguish miRNA target from its highly homologous family members (miR‐17 and let‐7 family members) by flexibly introducing a mismatch at the specific site of crRNA, no matter the single different base locates at the 5′ or 3′ end of the target (miR‐17 vs miR‐106a, let‐7b vs let‐7c). Moreover, the introduction of “light‐switch” molecule [Ru(phen)_2_dppz]^2+^ make the platform avoid tedious electrode modification and washing processes, thereby simplify experimental procedure, lower testing cost, and improve the reproduction of the results. Analysis of miRNA from raw cell lysates also demonstrates the platform holds a great potential for molecular diagnosis.

## 4 Experimental Section

##### Reagents and Apparatus

All the sequences used in this study were displayed in Tables S1 and S2 (Supporting Information). The DNA oligos were purchased from Sangon Biological Co., Ltd. (Shanghai, CN). All target miRNAs, RNA reporters (FQ5U) modified with fluorescent (FAM) and quencher (BHQ1) at 5′ and 3′‐end, respectively, deoxynucleotide (dNTP) mix, RNase‐free water, RNase inhibitor, RNAiso for small RNA, DNase I and its reaction buffer, and qRT‐PCR Kit were ordered from Takara Biotechnology Co., Ltd. (Dalian, CN). The various enzymes used in the experiments including T7 RNA polymerase, Vent (exo‐) DNA polymerase, NEase Nt. BstNBI, and T4 polynucleotide Kinase and their corresponding reaction buffer were all purchased from New England Biolabs (Beijing, CN). TPrA (≥98%) was ordered from Sigma‐Aldrich (St. Louis, MO). [Ru(phen)_2_dppz](PF_6_)_2_ was synthesized and characterized by Prof. Caiping Tan (Sun Yat‐Sen University).^[^ ^22^ ^]^

Real‐time fluorescence monitoring was executed via the CFX Connect real‐time PCR detection system (Bio‐Rad, CA). A transistor–transistor logic was used to amplify and differentiate the ECL signal. A multifunction acquisition card (PCI‐1751, Advantech, Taiwan, China) was employed to quantify the collected signals.

##### Expression and Purification Processes of LbuCas13a

Prof. Yanli Wang (Institute of Biophysics, Chinese Academy of Sciences, Beijing, CN) denoted the pET‐Sumo‐LbuCas13a expression vector. The LbuCas13a protein used in this study was expressed and purified according to previous reporter.^[^ ^11^ ^]^ In brief, *E. coli* Rosetta2 (DE3) cells with the transfected vector were cultured overnight in TB medium at 37 °C. Then 0.1 × 10^−3^ m IPTG (Sigma) was introduced to induce LbuCas13a expression at 16 °C overnight. After that, the DE3 cells were centrifuged and sonically lysed. Then centrifuged the lysate, and added the obtained supernatant into the Ni Sepharose (GE Healthcare) for purification. The protein bound to the column was eluted with elution buffer (with 250 × 10^−3^ m imidazole). Subsequently, Ulp1 protease was applied to remove the His6‐Sumo tag of the protein products, which then passed through the heparin column (GE Healthcare) for purification. Finally, the protein was eluted and concentrated to 2 mg mL^−1^ in storage buffer (with 50% glycerol). Then the protein was stored at −80 °C before use.

##### In Vitro Transcription and Purification of crRNA

A double‐stranded DNA (dsDNA) with a T7 promotor was used to transcribe crRNA in vitro. The template was formed by annealing two complementary DNA sequences from 95 to 25 °C in 1 × PCR buffer. The transcription reaction system with a total volume of 30 µL included 1 × T7 RNA polymerase reaction buffer, 2 × 10^−3^ m each NTP, 150 U T7 RNA polymerase, 30 U RNase inhibitor, and 1.2 ng DNA template, incubated at 37 °C overnight. Then DNase I was added to digest the DNA template. The RNA products were purified by RNA clean Kit (Tiangen) and quantified by Nanodrop 2000 UV−vis spectrophotometer. The purified crRNA was stored at −80 °C before use.

##### Cas13a Cleavage Assay

LbuCas13a and crRNA with a final concentration of 10 × 10^−9^ m were incubated in 1 × PCR buffer at 37 °C for 10 min. The 10 µL Cas13a reaction system contains 10 × 10^−9^ m Cas13a/crRNA complex, 50 × 10^−9^ m PT, and different concentrations of miRNA. Finally, the mixed solution was incubated at 37 °C for 30 min. After that, 0.2 U T4 PNK and 1 × T4 PNK reaction buffer were added into the solution to phosphorylate the 3′‐end of the cleaved PT, which can effectively initiate the following EXPAR. The reaction was carried out at 37 °C for 30 min.

##### EXPAR Assay

The EXPAR system was completed with two separated solutions, solution A and B. Wherein, the solution A contained 0.1 × 10^−6^ m of the template, 250 × 10^−6^ m dNTP mix, and 1.2 µL of the cleaved products; The solution B contained 0.4 U µL^−1^ NEase Nt.BstNBI, 0.05 U µL^−1^ Vent (exo‐) DNA polymerase, 1 × Thermopol buffer, and 1 × NEBuffer 3.1. Then the Part A and Part B were immediately mixed to a total volume of 10 µL and incubated at 55 °C for 30 min.

##### Cell Culture, Small RNA Extraction from Cells, and Recovery Experiments

All the cell lines used in this study were cultured in dulbecco's modified eagle medium with 10% fetal calf serum. The total small RNA was extracted using the small RNA extraction kit (Takara). The obtained total small RNA was quantified by Nanodrop 2000 and stored at −80 °C.

Briefly, 1 mL LO2 (≈5.2 × 10^6^) cells were centrifuged and resuspended in 500 µL 1 × PBS buffer. This process was repeated 2–3 times to wash away the cell culture medium. Then the cells were lysed at 95 °C for 5 min, and rapidly cooled on ice for 3 min. After that, different concentrations of miR‐17 (100 × 10^−12^ m, 1 × 10^−12^ m, 100 × 10^−15^ m, 10 × 10^−15^ m) were, respectively, added into 1 µL cell lysates and analyzed by PECL‐CRISPR.

##### Fabrication of the pBPE and Assay Procedures of pBPE‐ECL Sensor

The fabrication of pBPE was based on previous reports.^[^ ^15b^ ^]^ In brief, it mainly includes two steps. Step 1: Print the bipolar electrode and the driving electrodes on the paper by screen printing using conductive carbon ink; Step 2: The formation of a hydrophilic channel by wax printing.

##### A Detailed Assay Procedure of pBPE‐ECL is Described Below

The pBPE‐ECL chip was located in a 3D printing support. The 25 µL of mix containing 15 µL EXPAR products, 1 µL 0.5 × 10^−3^ m [Ru(phen)_2_dppz]^2+^, and 9 µL 70 × 10^−3^ m TPrA, which was prepared in 0.01m PBS (pH 7.4), was added onto the pBPE. A driving voltage (14 V) is provided by an external DC power supply connected to the driving electrode. After 10 s to ensure that the electrode is completely immersed in the solution, the ECL signal was measured using PMT. The PMT (MP‐962, PerkinElmer, Wiesbaden, Germany) voltage was 850 V.^[^ ^15b^ ^]^

## Conflict of Interest

The authors declare no conflict of interest.

## Supporting information

Supporting Information
