# Supplementary material for: CRISPR/Cas13a Powered Portable Electrochemiluminescence Chip for Ultrasensitive and Specific MiRNA Detection
Source: Adv Sci (Weinh). 2020 May 27;7(13):1903661. doi: 10.1002/advs.201903661 (PMC7341088; doi:10.1002/advs.201903661)
Supplement: Supplementary file 1 — Supporting Information [file ADVS-7-1903661-s001.pdf]

Supplementary Information

CRISPR/Cas13a Powered Portable Electrochemiluminescence Chip for Ultrasensitive and Specific MiRNA Detection

Ting Zhou[†], *Ru Huang*[†,*], *Mengqi Huang, Jinjin Shen, Yuanyue Shan, Da Xing**

Ting Zhou, Dr. R. Huang, Mengqi Huang, Jinjin Shen, Yuanyue Shan, Prof. D. Xing
MOE Key Laboratory of Laser Life Science & Institute of Laser Life Science, College of Biophotonics, South China Normal University, Guangzhou 510631, China
Email: xingda@scnu.edu.cn; huangru@scnu.edu.cn
[†] These authors contributed equally to this work.

Table of Contents

Table of Contents…………………………….…………………………………….I

1. Characterization of the pBPE-ECL device……………………………………………2

2. Purity and activity analysis of LbuCas13a……………………………………….3

3. Michaelis-Menten analysis of *trans*-cleavage activity of LbuCas13a on two rU based DNA probe…………………………………………………..…………4

4. Analysis of the effect of T4 PNK for PECL-CRISPR……………..……………5

5. Optimization of the reaction conditions……………………………………………6

6. Real-time fluorescence assay of CAS-EXPAR…….……………..……………8

7. Comparison of the LOD of Cas13a/crRNA and CAS-EXPAR by real-time fluorescence monitoring……………….……………….….…………..…………9

8. Quantitative real-time PCR experiments…………………………………………10

Table S1. Sequences for PECL-CRISPR..…………………………………………12

Table S2. Sequences for stem-loop RT-PCR………………………………………12

Table S3. The Ct value of stem-loop RT-PCR for various cell lines….……………13

REFERENCES……………………………………………………………....13

1. Characterization of the pBPE-ECL device.

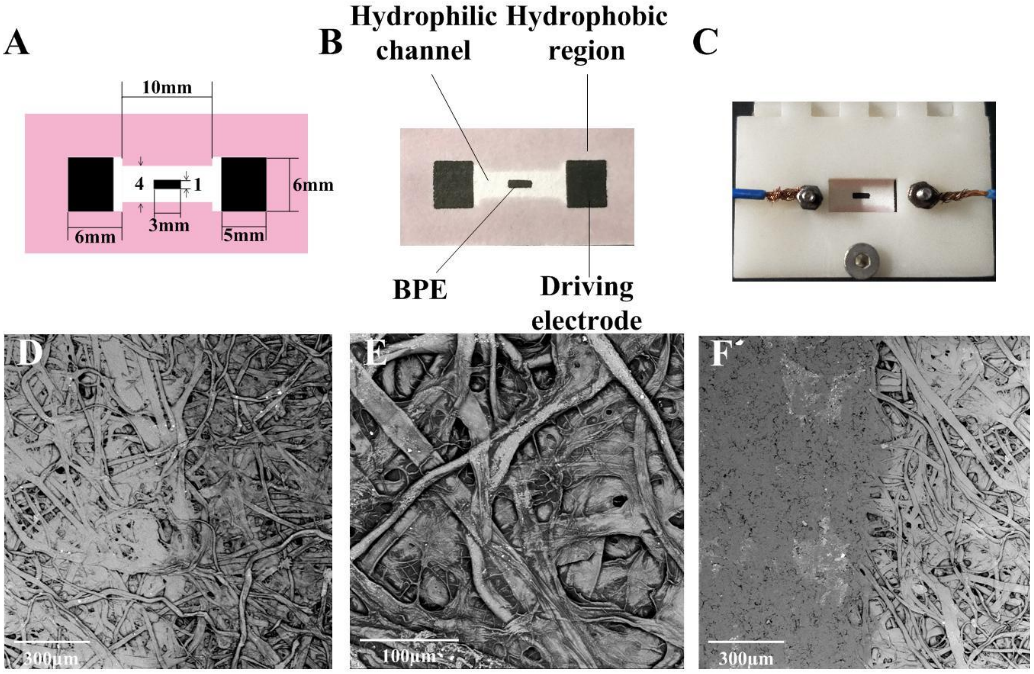

Figure S1. Characterization of the pBPE-ECL device. (A) Size of each part of the pBPE. (B) Photo of a screen-printed pBPE unit. The surrounding red region is the hydrophobic area made by wax printing, and the white part in the middle is the hydrophilic channel. The black parts that made by screen-print are a BPE (in the center) and two driving electrodes (at the two poles of the BPE). (C) Photo of an assembled pBPE chip. SEM images of (D) the boundary of wax pattern: left is pure paper, right is wax-printed paper; (E) front face of wax-penetrated paper; (F) the boundary between the carbon ink-screen-printed paper and the pure paper.

2. Purity and activity analysis of LbuCas13a.

The LbuCas13a was expressed from pET-Sumo-LbuCas13a expression vector transfected *E. coli* Rosetta2 (DE3) cells and purified by a series purification processes. The purity of the LbuCas13a protein was analyzed by SDS-PAGE and Coomassie blue staining. Moreover, the *trans* cleavage activity of Cas13a/crRNA was tested by real-time fluorescence monitoring.

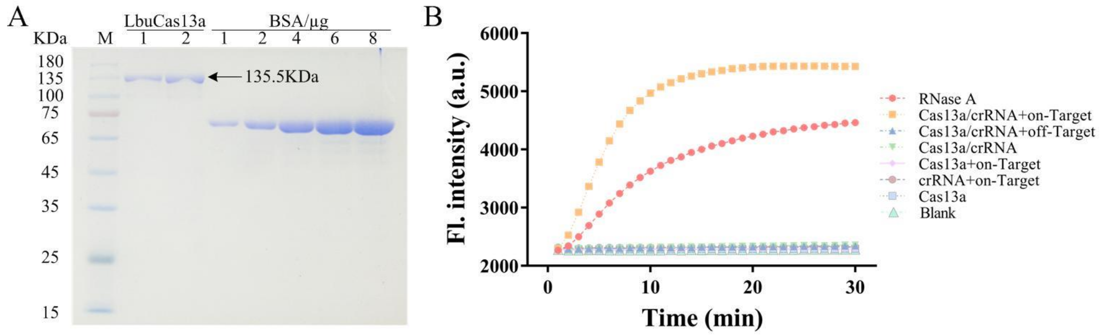

Figure S2. Purity and activity analysis of LbuCas13a. (A) SDS-PAGE results of purified LbuCas13a. (B) Fluorescent Assays of LbuCas13a activity. RNase A added sample was used as positive control. a.u., arbitrary units.

3. Michaelis-Menten analysis of *trans*-cleavage activity of LbuCas13a on two rU based DNA probe.

In order to test whether the *trans*-cleavage activity of Cas13a would be affected by the number of rU in the substrate, a reporter probe (length of 5 nt) named FQ2U was designed to have two rU in the middle, as well as a FAM dye and a BHQ1 quencher at the two ends respectively. Different concentrations of FQ2U (0.001, 0.01, 0.1, 0.2, 0.5, 1, 2, 5, and 10 μM) were incubated with 10 nM LbuCas13a/crRNA complex and 1 nM miR-10b, and tested by real-time fluorescence monitoring. The results were shown in **Figure S3**. The Michaelis constant of LbuCas13a (K_m) was determined by plotting initial velocity (V_0) as a function of substrate concentration ([S]). The Michaelis-Menten equation can be obtained according to the equation of $V_0=(V_{max}[S])/(K_m+[S])$, wherein V_{max} is maximum reaction rate. The turnover number of LbuCas13a (k_{cat}) was calculated by the equation of $k_{cat}=V_{max}/E_t$ (E_t indicating the concentration of LbuCas13a is 10 nM) as ~204. The catalytic efficiency k_{cat}/K_m was 0.77854×10^8 s^{-1} M^{-1}, which is >1400-fold lower than using five consecutive rU as the *trans*-cleavage substrate of LbuCas13a (k_{cat}/K_m of $1.0^9 \times 10^9$ s^{-1} M^{-1}, E_t was 0.01 nM) that we reported previously[1]. Overall, decreasing the rU number of the DNA oligo can significantly lower the *trans*-cleavage efficiency of LbuCas13a/crRNA.

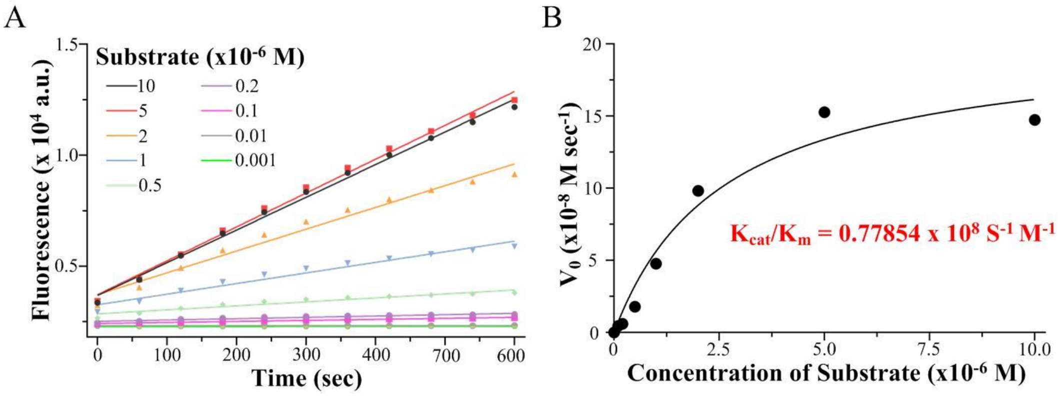

Figure S3. (A) Plot of real-time fluorescence monitoring results for miR-10b with different concentrations of FQ2U reporter (1 nM ~ 10 μM). The concentration of effective LbuCas13a/crRNA complex and miR-10b were 10 nM and 1 nM, respectively. (B)

Characterization of LbuCas13a *trans*-cleavage activity on two rU based DNA probe by Michaelis-Menten plot.

4. Analysis of the effect of T4 PNK for PECL-CRISPR.

We attempted to use LbuCas13a cleaved pre-primer to hybridize with a cyclized padlock probe and execute rolling circle amplification (RCA). The PAGE result (**Figure S4**) showed that the cleaved pre-primer cannot trigger RCA reaction directly. According to previous report, Cas13 *trans*-cleavage activity generates two fragments: one has a hydroxylated at its 5'-end, the other possesses a 2',3'-cyclic phosphate at its 3'-end[2]. Considering the 2',3'-cyclic phosphate at the 3'-end of the cleaved pre-primer may hinder the polymerization reaction, T4 PNK was used to transform the 2', 3'-cyclic phosphate group into 3'-phosphate group. The PAGE image of **Figure S4** showed that only T4 polynucleotide kinase (T4 PNK)-treated Cas13a-cleaved pre-primer can induce obvious RCA products, which indicated that T4 PNK mediated phosphorylation is the key to initiate DNA polymerization reaction.

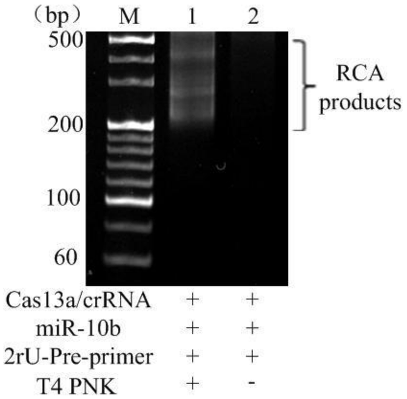

Figure S4. PAGE analysis of the RCA products with or without T4 PNK. M is marker.

5. Optimization of the reaction conditions.

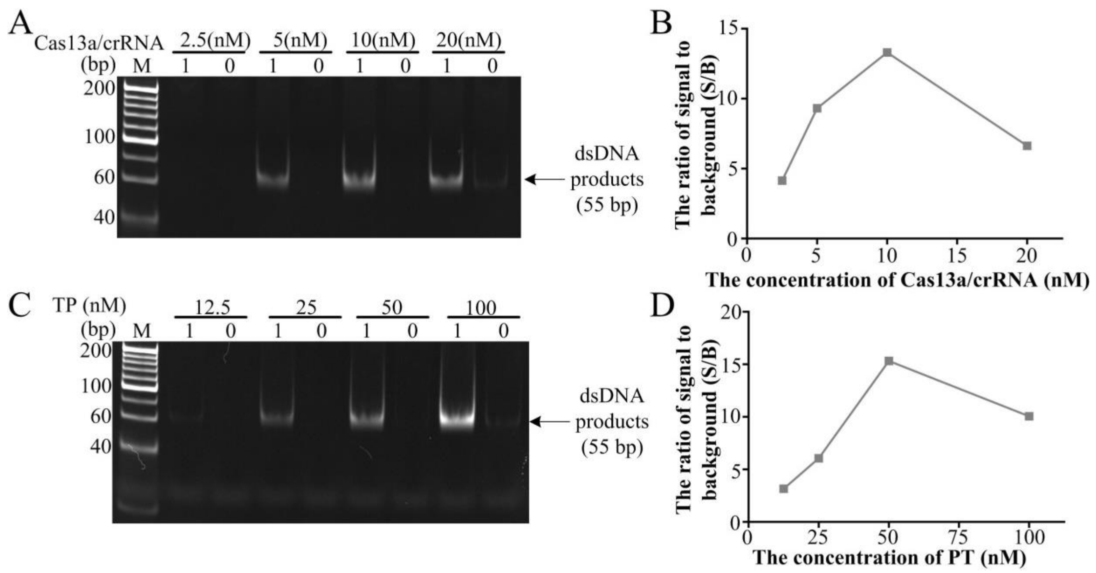

Figure S5. Optimization of the reaction conditions by non-denaturing PAGE assays. Imaging results (A) and gray analysis (B) of EXPAR with different Cas13a/crRNA concentration (2.5 nM, 5 nM, 10 nM, 20 nM). 1 represents the test sample with 1 nM miR-17; 0 represents the negative control sample. Imaging results (C) and gray analysis (D) of EXPAR with different concentrations (12.5 nM, 25 nM, 50 nM, 100 nM) of pre-primer (PT).

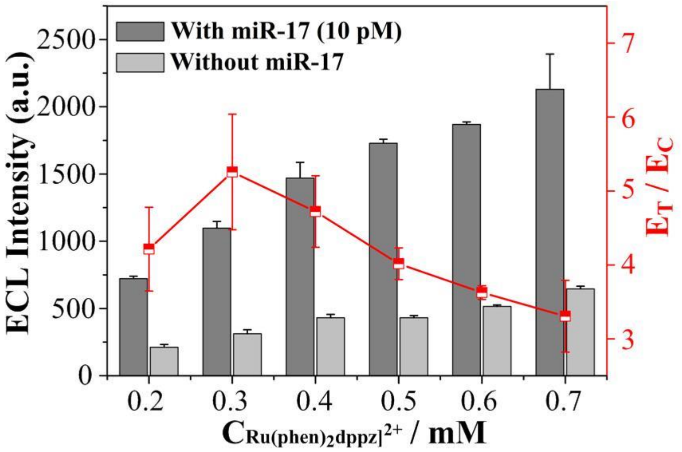

Figure S6. Optimization of $[Ru(phen)_2dppz]^{2+}$ concentration. The $[Ru(phen)_2dppz]^{2+}$ concentration were 0.2, 0.3, 0.4, 0.5, 0.6 and 0.7 mM, respectively. E_T represents the ECL signal intensity of the sample with 10 pM miR-17, and E_C was the corresponding control sample without miR-17.

6. Real-time fluorescence assay of CAS-EXPAR.

The CAS-EXPAR was analysed by the real-time fluorescence assay with 1 nM miR-17 as shown in **Figure S7**. The result reflected the EXPAR reaction requires about 20 min to complete.

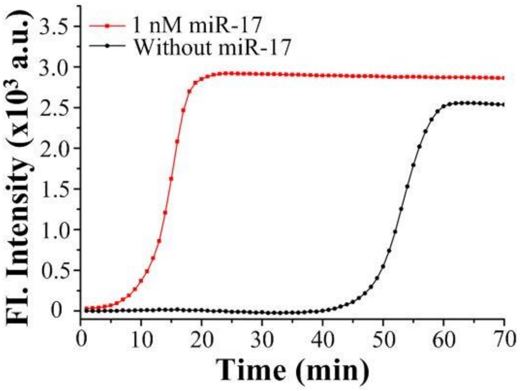

Figure S7. Real-time fluorescence assay of CAS-EXPAR.

7. Comparision of the LOD of Cas13a/crRNA and CAS-EXPAR by Real-time Fluorescence Monitoring

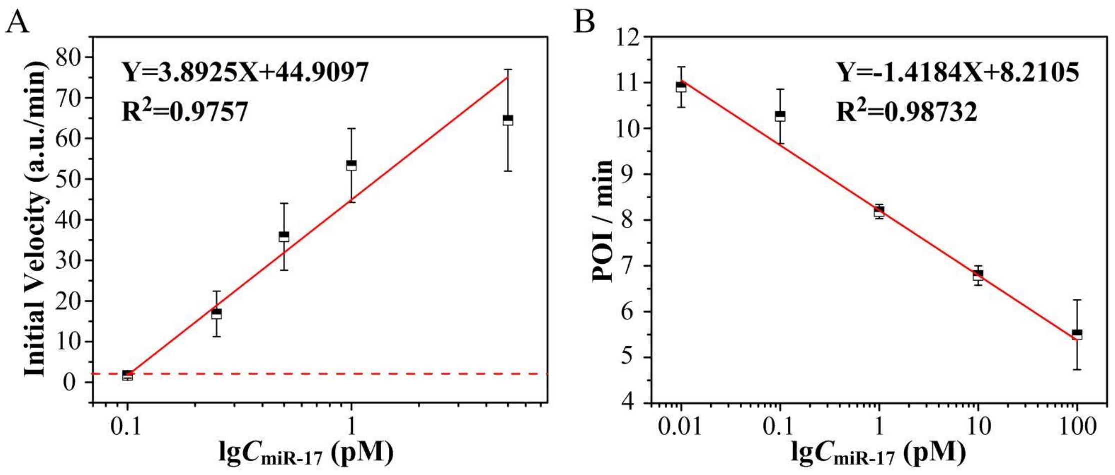

Figure S8. (A) Linear analysis of Cas13a/crRNA mediated miR-17 detection. The concentration of target miR-17 were 5 nM, 1 nM, 500 pM, 250 pM and 100 pM, respectively. (B) Linear analysis of CAS-EXPAR for miR-17 detection. The concentration of target miR-17 were 100 pM, 10 pM, 1 pM, 100 fM, and 10 fM, respectively.

8. Quantitative real-time PCR experiment

The reverse transcriptase (RT) reactions contained the total small RNA (20–200 nt, 400 ng, extracted from cell lines), 0.1 μM stem-loop RT primer, 1×RT buffer, 10 mM each of dNTPs, 10 U/μL M-MLV reverse transcriptase and 1U/μL RNase inhibitor, and the total volume is 10 μL. The RT reaction was carried out in the thermocycler at 37 °C for 60 min and 95 °C for 3 min. Real-time PCR was performed using SYBR Green I fluorescence dye. The 20 μL real-time PCR reaction contained 1 μL of RT product, 1× SYBR Green qPCR Mix, 0.8 μM forward primer and 0.4 μM reverse primer. The reaction was carried out in Applied Biosystems real-time PCR at 95 °C for 3 min and 40 cycles of 95 °C for 5 s and 60 °C for 30 min. snRNA U6: G U C C C U U C G G G G A C A U C C G A U A A A A U U G G A A C G A U A C A G A G A A G A U U A G C A U G G C C C C U G C G C A A G G A U G A C A C G C A C A A A U C G A G A A A U G G U C C A A A A U U U U.

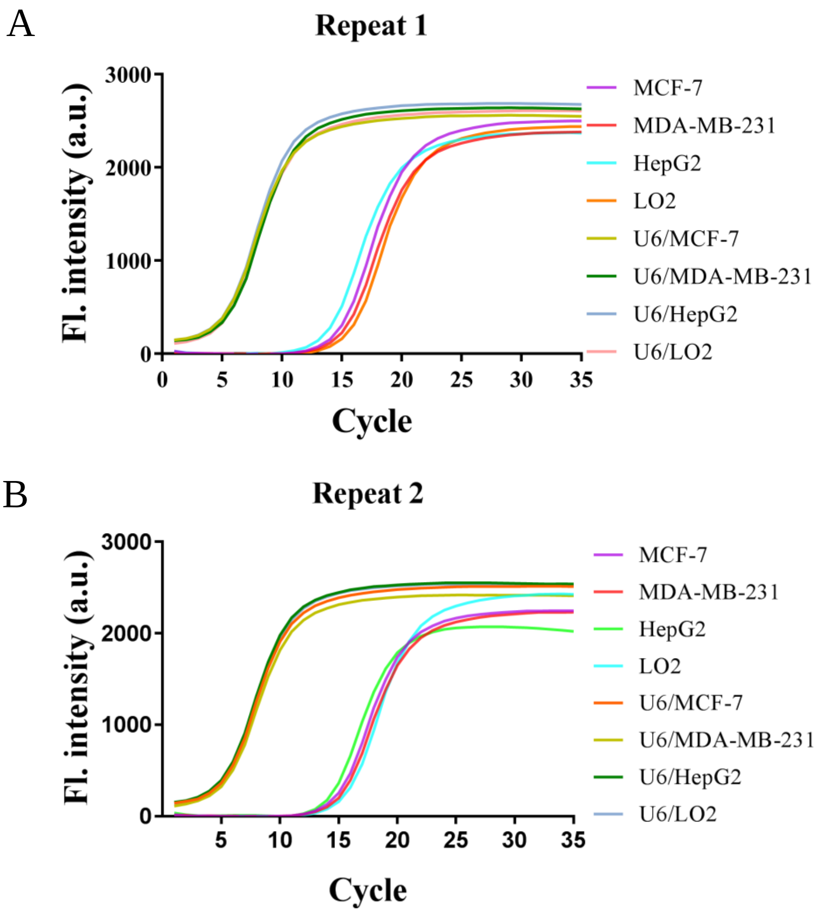

C

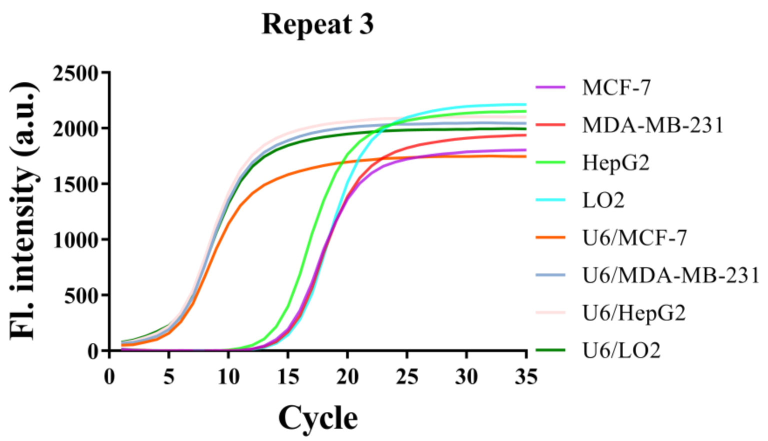

Figure S9. Quantitative real-time PCR experiment. A-C. The real-time reverse transcription PCR for miRNA17 and U6 small nuclear RNA (snRNA) detection in various cell lines. U6 was employed as the universal endogenous control and the relative expression was calculated by the equation: Fold change=$2^{-\Delta\Delta Ct}$.

Table S1. Sequences for PECL-CRISPR.

Name	Sequence(5'-3')
miR-17	CAAAGUGCUUACAGUGCAGGUAG
miR-106a	AAAAGUGCUUACAGUGCAGGUAG
miR-20a	UAAAGUGCUUAUAGUGCAGGUAG
miR-20b	CAAAGUGCUCAUAGUGCAGGUAG
miR-10b	UACCCUGUAGAACCGAAUUUGUG
miR-21	UAGCUUAUCAGACUGAUGUUGA
miR-155	UUAAUGCUAAUCGUGAUAGGGGU
let-7a	UGAGGUAGUAGGUUGUAUAGUU
let-7b	UGAGGUAGUAGGUUGUAUGGUU
let-7c	UGAGGUAGUAGGUUGUGUGGUU
FQ5U	FAM-rUrUrUrUrU-BHQ1
crR-17	GACCACCCCAAAAAUGAAGGGGACUAAAACCUACCUGCACUGUAAGCACUUUG
cr17-M16	GACCACCCCAAAAAUGAAGGGGACUAAAACCACUGUAAGCAGUUUG [b)]
cr7b	GACCACCCCAAAAAUGAAGGGGACUAAAACAACCACACAACCUACUACCU
cr7b-M4	GACCACCCCAAAAAUGAAGGGGACUAAAACAACGACACAACCUACUACCU [b)]
cr7b-M8	GACCACCCCAAAAAUGAAGGGGACUAAAACAACCACAGAACCUACUACCU [b)]
Pre-trigger	TTGGATGGATATTGT-rUrU-CATA - C3 spacer [a)]
Template	AACTATCAACAATATCCATCCAAACAGACTCAAACTATCAACAATATCCATCCAA - C3 spacer

[a)] rU represents uracil ribonucleotide. Others are all deoxyribonucleotides. [b)]Red fonts represent the mismatch sites.

Table S2. Sequences for stem-loop RT-PCR.

Note	Sequence (5'-3')
RT hairpin	GTCGTATCCAGTGCAGGGTCCGAGGTATTCGCACTGGATACGACTCAACA
Forward primer	GCCCGCTAGCTTATCAGACTGATG
Reverse primer	GTGCAGGGTCCGAGGT
U6 Forward primer	ATTGGAACGATACAGAGAAGATT
U6 Reverse primer	GGAACGCTTCACGAATTTG

Table S3. The Ct value of stem-loop RT-PCR for various cell lines.

Note	$Ct_{miRNA17}$	CtU6	−ΔΔCt	Fold change
LO2-1	17.03	6.19	-	1
MCF-7-1	16.51	6.23	0.56	1.47
MDA-MB-231-1	16.05	5.97	0.76	1.69
HepG2-1	15.21	5.91	1.55	2.93
LO2-2	17.03	6.08	-	1
MCF-7-2	16.64	6.27	0.58	1.49
MDA-MB-231-2	16.34	6.08	0.69	1.61
HepG2-2	15.74	5.93	1.13	2.54
LO2-3	16.53	6.39	-	1
MCF-7-3	16.35	6.56	0.34	1.275
MDA-MB-231-3	16.17	6.88	0.85	1.8
HepG2-3	15.01	6.3	1.43	2.69

References:

1. Y. Shan, X. Zhou, R. Huang, D. Xing, *Anal. Chem.* **2019**, *91*, 5278.
2. J. S. Gootenberg, O. O. Abudayyeh, M. J. Kellner, J. Joung, J. J. Collins, F. Zhang, *Science* **2018**, *360*, 439.
